# One amino acid makes the difference: the formation of *ent*-kaurene and 16α-hydroxy-*ent-*kaurane by diterpene synthases in poplar

**DOI:** 10.1186/s12870-015-0647-6

**Published:** 2015-10-28

**Authors:** Sandra Irmisch, Andrea T. Müller, Lydia Schmidt, Jan Günther, Jonathan Gershenzon, Tobias G. Köllner

**Affiliations:** Max Planck Institute for Chemical Ecology, Hans-Knöll-Strasse 8, D-07745 Jena, Germany

**Keywords:** *Populus trichocarpa*, Diterpene synthases, *Ent*-kaurene, 16α-hydroxy-*ent-*kaurane, Gene duplication, Gibberellin biosynthesis

## Abstract

**Background:**

Labdane-related diterpenoids form the largest group among the diterpenes. They fulfill important functions in primary metabolism as essential plant growth hormones and are known to function in secondary metabolism as, for example, phytoalexins. The biosynthesis of labdane-related diterpenes is mediated by the action of class II and class I diterpene synthases. Although terpene synthases have been well investigated in poplar, little is known about diterpene formation in this woody perennial plant species.

**Results:**

The recently sequenced genome of *Populus trichocarpa* possesses two putative copalyl diphosphate synthase genes (*CPS*, class II) and two putative kaurene synthase genes (*KS*, class I), which most likely arose through a genome duplication and a recent tandem gene duplication, respectively. We showed that the *CPS*-like gene *PtTPS17* encodes an *ent*-copalyl diphosphate synthase (*ent*-CPS), while the protein encoded by the putative *CPS* gene *PtTPS18* showed no enzymatic activity. The putative kaurene synthases PtTPS19 and PtTPS20 both accepted *ent*-copalyl diphosphate (*ent*-CPP) as substrate. However, despite their high sequence similarity, they produced different diterpene products. While PtTPS19 formed exclusively *ent*-kaurene, PtTPS20 generated mainly the diterpene alcohol, 16α-hydroxy-*ent-*kaurane. Using homology-based structure modeling and site-directed mutagenesis, we demonstrated that one amino acid residue determines the different product specificity of PtTPS19 and PtTPS20. A reciprocal exchange of methionine 607 and threonine 607 in the active sites of PtTPS19 and PtTPS20, respectively, led to a complete interconversion of the enzyme product profiles. Gene expression analysis revealed that the diterpene synthase genes characterized showed organ-specific expression with the highest abundance of *PtTPS17* and *PtTPS20* transcripts in poplar roots.

**Conclusions:**

The poplar diterpene synthases PtTPS17, PtTPS19, and PtTPS20 contribute to the production of *ent*-kaurene and 16α-hydroxy-*ent-*kaurane in poplar. While *ent*-kaurene most likely serves as the universal precursor for gibberellins, the function of 16α-hydroxy-*ent-*kaurane in poplar is not known yet. However, the high expression levels of *PtTPS20* and *PtTPS17* in poplar roots may indicate an important function of 16α-hydroxy-*ent-*kaurane in secondary metabolism in this plant organ.

**Electronic supplementary material:**

The online version of this article (doi:10.1186/s12870-015-0647-6) contains supplementary material, which is available to authorized users.

## Background

Terpenoids are found in almost all life forms fulfilling a wide array of important functions. With over 60,000 different structures described at present, terpenoids represent the largest and structurally most diverse group of natural products [1]. This biodiversity arises from only a few prenyl diphosphate precursors. Terpene synthases (TPSs), the key enzymes of terpene metabolism, accept these precursors as substrates and convert them into monoterpene (C_10_), sesquiterpene (C_15_), or diterpene (C_20_) products, usually olefins and alcohols. Due to their high volatility, many monoterpenes and sesquiterpenes are main constituents of vegetative or floral scents thereby playing important roles in plant-insect interactions or intra- and inter-plant communication [2, 3]. Diterpenoids are in general less volatile, but also often function in the interactions of plants with other organisms. They are, for example, major constituents in the resin of different conifer species defending against shoot-infesting insects [4, 5]. Rice (*Oryza sativa*) has a large number of diterpenoid phytoalexins possessing antifungal activities [6] and in maize the diterpenoid kauralexins were shown to be involved in antiherbivore and antifungal defense [7]. Apart from this important function in plant defense, some diterpenoids are essential for plants. *Ent*-kaurene, for example, is the precursor for the gibberellins, which represent an important group of plant hormones involved in various physiological processes (recently reviewed in [8]).

Geranylgeranyl diphosphate (GGPP) is the universal precursor for all plant diterpenes. Different combinations of diterpene synthases and P450 enzymes lead to the production of the great diversity of about 12,000 diterpenoids known to date with the biggest group being labdane-related compounds [9]. The formation of labdane-related diterpenes is mediated by the action of class II and class I diterpene synthases [10]. Class II diterpene synthases accept GGPP as substrate and catalyze the formation of bicyclic prenyl diphosphates. They are characterized by a highly conserved DxDD motif which mediates the initial protonation of the substrate [11]. The bicyclic prenyl diphosphates can be further converted by class I diterpene synthases which possess characteristic DDxxD and a NSE/DTE motifs. Class I enzymes catalyze the metal ion-dependent ionization of the substrate, resulting in the formation of a carbocation which can undergo further cyclization and rearrangement reactions [12]. The carbocationic reaction mechanism of the class I enzymes leads to the large structural variety of the diterpenes [13].

The biosynthesis of the gibberellins has been quite well investigated. Their formation starts with the conversion of GGPP into *ent*-copalyl diphosphate (CPP) catalyzed by a class II enzyme, *ent*-CPP synthase (CPS). Subsequently, a class I enzyme, kaurene synthase (KS), converts *ent*-CPP to *ent*-kaurene via a complex bicyclization and ring rearrangement reaction (recently reviewed in [8, 9]). While higher plants usually possess monofunctional CPS and KS enzymes [13], the moss *Physcomitrella patens* possesses a bifunctional CPS/KS containing two active sites converting GGPP directly into *ent*-kaurene [14]. In contrast to Arabidopsis which possesses only individual *CPS* and *KS* genes, both involved in gibberellin biosynthesis [15–17], the *CPS* and *KS* gene families have expanded in other plant species. Rice, for example, contains four *CPS*/*CPS*-like genes and eleven *KS*/*KS*-like genes involved in the production of a large variety of different labdane-type diterpenes [6, 18, 19]. Here, class I terpene synthases not mediating *ent*-kaurene formation but generating other labdane-related diterpenes are called kaurene synthase-like enzymes (KSL) [19].

The *TPS* gene family in *Populus trichocarpa* has recently been characterized [20, 21]. However, the focus of this study was on mono- and sesquiterpene synthases and only one diterpene synthase, the geranyl linalool synthase PtTPS10, was described. In addition to *PtTPS10*, *P. trichocarpa* also contains two putative *CPS* and two putative *KS* genes [21] which were designated *PtTPS17*, *PtTPS18* and *PtTPS19*, *PtTPS20*, respectively. In the present study we investigated these genes and the encoded CPS and KS enzymes.

## Results

### Poplar possesses two putative copalyl diterpene synthase genes (*CPS*) and two putative kaurene synthase (like)- (*KS(L)*) genes

Besides the recently characterized geranyllinalool synthase gene *PtTPS10*, the poplar genome contains four additional genes (Potri.002G05210, Potri.005G210300, Potri.008G082400, and Potri.008G082700) encoding putative diterpene synthases [21]. A blast analysis revealed that Potri.002G052100 and Potri.005G210300 had high similarity to *CPS* genes from other plants while Potri.008G082400 and Potri.008G082700 were most similar to *KS* genes. We were able to amplify Potri.002G05210, Potri.005G210300, Potri.008G082400, and Potri.008G082700 from a cDNA pool attained from leaf buds, leaves, stems, and roots of *Populus trichocarpa* and the open reading frames obtained were designated *PtTPS17*, *PtTPS18*, *PtTPS19,* and *PtTPS20*, respectively.

**Fig. 1:**
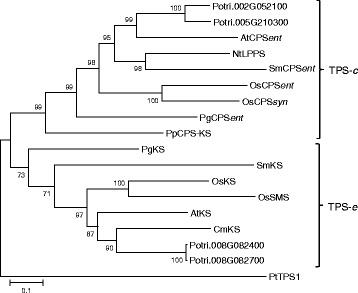
Phylogenetic tree of putative kaurene synthase-(like) enzymes (KS(L)) and copalyl diphosphate synthases (CPS). The phylogenetic relationship of putative KS(L) and CPS synthases from *P. trichocarpa* to KS(L) and CPS from other plant species is shown. The tree was inferred with the neighbor-joining method and *n* = 1000 replicates for bootstrapping. Bootstrap values are shown next to each node. TPS-*c* and TPS-*e,* represent established TPS subfamilies [13]. PtTPS1 was used as an outgroup. KS: *ent*-kaur-16-ene synthase, SMS: stemar-13-ene synthase, LPPS: 8-hydroxy-copalyl diphosphate synthase, CPS: copalyl diphosphate synthase, Nt: *Nicotiana tabacum,* Cm: *Cucurbita maxima,* At: *Arabidosis thaliana,* Os: *Oryza sativa*, Pg: *Picea glauca*, *Potri: Populus trichocarpa, Sm: Salvia miltiorrhiza, Pp: Physcomitrella patens*

**Fig. 2:**
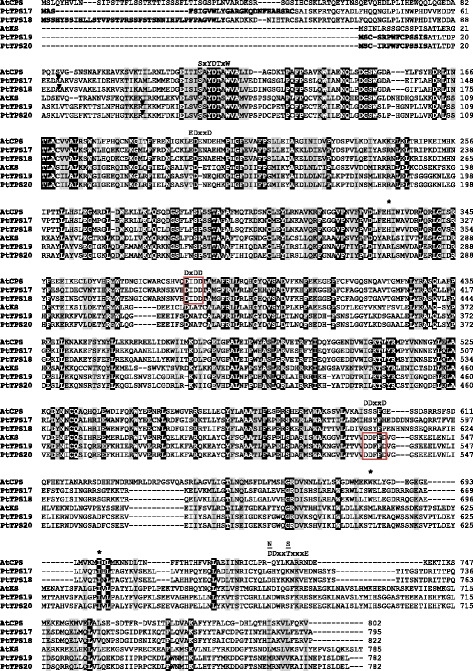
Amino acid sequence comparison of putative CPS and KS(L) from *P. trichocarpa* with characterized *ent*CPS and KS from *A. thaliana*. Identical amino acids are marked by black boxes and amino acids with similar side chains are marked by gray boxes. Conserved motifs are labeled and the highly conserved DxDD and DxxDD motifs are boxed red. Asterisks indicate amino acids important for regulation and product specificity. Predicted N-terminal signal peptides are bold and an arrow indicates the truncation site for heterologous expression. AtCPS (Q38802), *ent*-copalyl diphosphate synthase; AtKS (Q9SAK2), kaurene synthase of *Arabidopsis thaliana*

*PtTPS17* and *PtTPS18* share 89.4 % nucleotide similarity and are located on chromosome two and five, respectively, according to the available databases (www.phytozome.org). The high sequence similarity and the chromosomal locations of *PtTPS17* and *PtTPS18* indicate their origin through the recent genome duplication event described for poplar [22]. In a phylogenetic tree, the encoded proteins cluster together with characterized CPS proteins from other plants and are members of the TPS-*c* family (Fig. 1). Sequence motifs characteristic for class II TPS enzymes and important for CPS activity, such as the DxDD motif responsible for the initial protonation of the double bond and the EDxxD-like motif that coordinates the Mg^2+^ / diphosphate [13, 23], could be identified in both enzymes (Fig. 2). In addition, both proteins contained a conserved histidine residue that has been described to mediate sensitivity towards Mg^2+^ [24].

The close association of *PtTPS19* and *PtTPS20* on chromosome 8 and their high sequence similarity of 99.3 % indicate that these genes evolved through a recent tandem gene duplication event (Additional file 1: Figure S1). The encoded proteins belong to the TPS-e family (Fig. 1) and contain sequence motifs important for the activity of class I TPS enzymes, like the DDxxD motif and the NSE/DTE motif for the metal ion-dependent ionization of the prenyl diphosphate substrate (Fig. 2) [13]. The proteins are most likely monofunctional enzymes as none of them contained both class I and class II TPS features (Fig. 2).

A signal peptide prediction using different prediction programs revealed that PtTPS17, PtTPS18, PtTPS19, and PtTPS20 contain N-terminal transit peptides (Fig. 2, Additional file 1: Table S3). Although, regarding the subcellular targeting of the enzymes, the different prediction algorithms gave different results (Additional file 1: Table S3). However, targeting of the enzymes to the plastids is most likely as diterpene biosynthesis is known to be localized in the chloroplasts.

### PtTPS17 produces *ent*-CPP and PtTPS19 and PtTPS20 have KS and KSL enzyme activity, respectively

To determine the enzymatic function of the putative poplar CPS and KS(L) proteins, truncated versions lacking the predicted signal peptides but still containing the N-terminal SxYDTxW motif reported to be conserved in KS and CPS enzymes [25] were heterologously expressed in *Escherichia coli*. In addition, an *ent-CPS* (AtCPS*, Arabidopsis thaliana)*, a *syn-CPS* (OsCPS4, *Oryza sativa*, making *syn*-copalyl diphosphate*)* and a *n-CPS* (AgAS:D621A, *Abies grandis,* making normal copalyl diphosphate) were expressed to provide potential substrates for KS(L) enzymes. Assays were conducted using crude enzyme extracts or purified protein and contained either the individual poplar proteins PtTPS17-20 or combinations of those enzymes with the different CPS mentioned above.

**Fig. 3:**
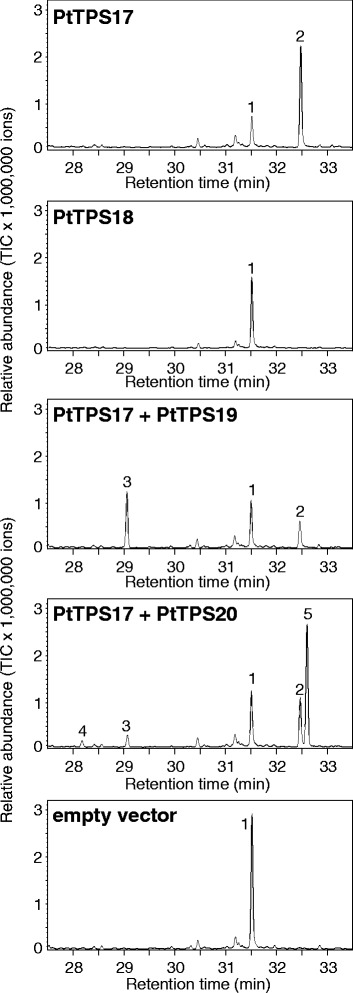
GC-MS analysis of diterpenoids produced by recombinant PtTPS17, PtTPS18, PtTPS19 and PtTPS20. The enzymes were expressed in *E. coli*, extracted, partially purified, and incubated with the substrate GGPP. Products were extracted with hexane and analyzed by GC-MS. 1, geranyllinalool; 2, copalol; 3, *ent*-kaurene; 4, *ent*-isokaurene; 5, 16α-hydroxy-*ent-*kaurane

**Fig. 4:**
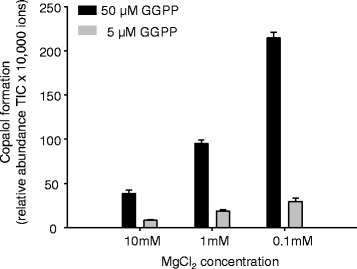
Sensitivity of PtTPS17 *ent*-CPP formation to Mg^2+^. The enzyme was expressed in *E. coli*, extracted, partially purified, and incubated with the substrate GGPP. The product CPP was hydrolyzed using HCl and extracted with hexane and analyzed by GC-MS

While no activity with GGPP could be observed for the putative KS(L) enzymes PtTPS19 and PtTPS20, neither alone nor in combinations with *syn*-CPS or *n*-CPS, diterpene product formation occurred when these enzymes were fed with GGPP in the presence of an *ent*-CPS. PtTPS19 converted *ent*-CPP into *ent-*kaurene and PtTPS20 converted this intermediate into 16α-hydroxy-*ent*-kaurane (86 %) and smaller amounts of *ent*-kaurene (8 %) and *ent-*isokaurene (6 %) (Fig. 3, Table 1). When PtTPS17 was incubated with GGPP, copalol was detected, as a result of the dephosphorylation of CPP. A comparison of the retention time of the copalol formed with those of authentic standards revealed that PtTPS17 produced either *ent*-CPP or *n*ormal-CPP (Additional file 1: Figure S2). However, the fact that PtTPS17 was able to support diterpene product formation when coupled with PtTPS19 or PtTPS20 confirmed that the enzyme mediated the formation of *ent*-CPP. Supplying PtTPS17 with different concentrations of Mg^2+^ did influence enzyme activity, with *ent*-CPP formation being higher at lower cofactor concentrations (Fig. 4). Despite the high sequence similarity to PtTPS17, no enzyme activity, neither with GGPP alone nor in combination with other CPS or KS, could be observed for PtTPS18 (Fig. 3). That a few amino acid mutations can affect enzyme activity has been shown for various terpene synthases (e.g. [26]). In all assays geranyllinalool formation could be detected, reflecting an unspecific dephosphorylation of the GGPP substrate. Attempts to verify enzyme activity in vivo by using crude protein extracts from poplar roots and leaves were not successful.

**Table 1:** Relative product formation of KS(L) enzymes

	*Ent*-isokaurene (%)	*Ent*- kaurene (%)	Hydroxy-*ent*-kaurane (%)
PtTPS19	0	100	0
PtTPS20_(T→M)_	0	100	0
PtTPS20	5.8 ± 1.7	8.1 ± 0.2	86.1 ± 1.5
PtTPS19_(M→T)_	5.8 ± 3.2	5.9 ± 1.1	88.3 ± 3.9
PtTPS19_(M→A)_	10.8 ± 2.5	6.9 ± 0.9	82.2 ± 3.4

The enzymes were expressed in *E. coli*, extracted, partially purified, and incubated with PtTPS17 and the substrate GGPP. Products were extracted with hexane and analyzed by GC-MS Means (*n* = 3) and standard errors (SE) are shown

### One amino acid determines the product specificity of PtTPS19 and PtTPS20

**Fig. 5:**
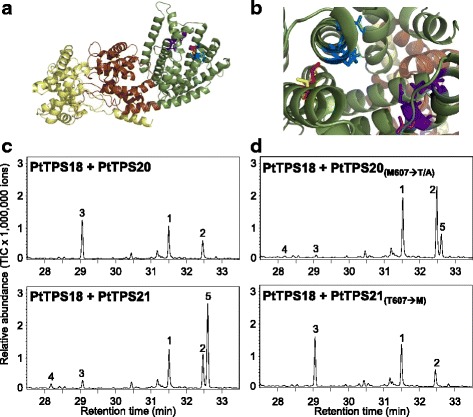
Substrate specificity of PtTPS19 and PtTPS20. **a** Model of PtTPS19 showing their three domain structure (yellow: γ-domain, brown: β-domain, green: α-domain). **b** Model of the aligned active sites of PtTPS19 and PtTPS20. The conserved DDxxD motif is shown as blue sticks and the NDxxTxxxE/DDxxSxxxE motif is represented by purple sticks. Met_607_ of PtTPS19 and Thr_607_ of PtTPS20, which influence product outcome, are depicted as red and yellow sticks, respectively. Product formation of wild type enzymes (**c**) and enzymes possessing one amino acid exchange (**d**). The enzymes were expressed in *E. coli*, extracted, partially purified, and incubated with PtTPS17 and the substrate GGPP. Products were extracted with hexane and analyzed by GC-MS. 1, geranyllinalool; 2, copalol; 3, *ent-*kaurene; 4, *ent*-isokaurene; 5, 16α-hydroxy-*ent-*kaurane

Although the PtTPS19 and PtTPS20 amino acid sequences were highly similar (99.1 %), their enzyme product profiles differed significantly. While PtTPS19 produced exclusively the diterpene hydrocarbon *ent-*kaurene, PtTPS20 mainly formed the diterpene-alcohol 16α-hydroxy-*ent-*kaurane (Fig. 3). To identify amino acids responsible for product specificity, homology-based structure models of PtTPS19 and PtTPS20 were constructed. Both models showed the three-domain structure (β, γ, and α domain) characteristic for the majority of plant DiTPS, with the catalytic site forming a deep pocket in the α domain (Fig. 5a,b; [23]). Only one amino acid differed in the active site of PtTPS19 compared to PtTPS20 (Fig. 2). While a methionine residue was present at position 607 in PtTPS19, the smaller, more polar threonine was situated at this position in PtTPS20 (Fig. 5b). Exchanging threonine 607 of PtTPS20 for methionine changed the product output of PtTPS20 completely. Instead of quenching the beyeran-16-yl cation by adding a water molecule and thus producing 16α-hydroxy-*ent*-kaurane, as observed for the wild type PtTPS20, the mutant enzyme catalyzed a deprotonation of the *ent*-kauranyl cation resulting in *ent-*kaurene formation comparable to PtTPS19 (Fig. 5d). Vice versa*,* the exchange of methionine 607 into a threonine in PtTPS19 resulted in a mutant able to produce mainly 16α-hydroxy-*ent-*kaurane and smaller amounts of *ent*-kaurene and *ent*-isokaurene in similar ratios as described for PtTPS20 (Fig. 5c, Table 1). The mutant PtTPS19 M_607_A produced also mainly 16α-hydroxy-*ent-*kaurane. However, exchanging the respective threonine 607 for alanine in PtTPS20 did not alter product specificity in comparison to the wild type enzyme (Table 1).

### *PtTPS17*-*20* are differentially expressed in poplar

**Fig. 6:**
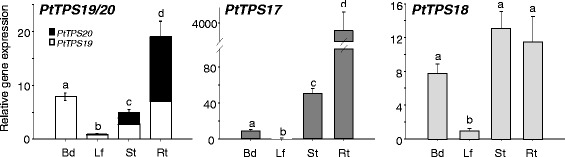
Transcript abundance of *PtTPS19/20, PtTPS17* and *PtTPS18* genes in different organs of *P. trichocarpa*. Gene expression in leaf buds (Bd), leaves (Lf), stem (St) and roots (Rt) was measured using qRT-PCR. *PtTPS19* to *PtTPS20* ratio was determined through repeated sequencing of amplicons. Means and standard errors are shown (*n* = 6). A one way ANOVA followed by a Holm-Sidak test was used to test for statistical significance. Different letters indicate significant differences between plant organs. *PtTPS19/20*: *F* = 140.549, *p* = <0.001; *PtTPS17*: *F* = 271.955, *p* = <0001; *PtTPS18*: *F* = 31.952, *p* = <0.001

To furthermore characterize the *CPS* and *KS(L)* synthase genes, we measured their transcript abundance in leaf buds, leaves, stems and roots of *P. trichocarpa* using quantitative (q)RT-PCR. Comparing the four different poplar organs, the transcript levels of the analyzed genes significantly differed (Fig. 6). The highest gene expression of *PtTPS17* and *PtTPS19/20* was found in roots, showing about 3500-fold and 20-fold higher expression, respectively, compared to leaves. A quite strong transcript accumulation was also found for *PtTPS17* in the stem (about 50-fold higher compared to leaves) and for *PtTPS19/20* in leaf buds and stems (about 8-fold and 5-fold higher, respectively, compared to leaves). All analyzed genes had the lowest transcript abundance in leaves. While *PtTPS17* and *PtTPS19/20* expression levels varied between the different poplar organs, *PtTPS18* showed a similar expression in leaf buds, stems and roots with about 10-fold higher transcript abundance compared to leaves (Fig. 6). The smaller c_q_-values for *PtTPS19/20* in comparison to those from *PtTPS17/18* indicate that *PtTPS19/20* were in general more strongly expressed than *PtTPS17* and *PtTPS18* (Additional file 1: Table S1). Due to their high nucleotide sequence similarity of about 99.4 %, it was not possible to distinguish between *PtTPS19* and *PtTPS20* in the qRT-PCR. However, repeated sequencing of cloned qRT-PCR products revealed that *PtTPS20* was not present in leaf buds, only slightly expressed in leaves (15.0 ± 6.2 % of total amplicons) and more strongly expressed in stems and roots (44.4 ± 9.6 and 63.2 ± 13.9 % of total amplicons, respectively, Fig. 6).

**Fig. 7:**
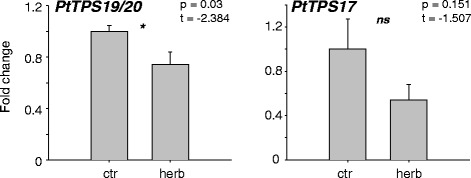
Transcript abundance of *PtTPS19/20* and *PtTPS17* in herbivore-damaged (herb) and undamaged control (ctr) leaves of *P. trichocarpa*. Caterpillars were allowed to feed for 24 h on apical LPI3 (leaf plastochron index 3) leaves. Gene expression was determined by qRT-PCR. Means and standard errors are shown (*n* = 5). The student’s *t*-test was used to test for statistical significance. Asterisks indicate a significant difference between herbivore-infested and untreated control leaves. ctr, control treatment; herb, herbivory

Since it is known that herbivory often induces the expression of terpene synthase genes involved in plant defense [21, 27], we measured the transcript accumulation of *PtTPS17/19/20* in undamaged and herbivore-damaged poplar leaves to investigate a putative role for these genes in defense against caterpillars. However, the qRT-PCR results showed that gene expression of *PtTPS17/19/20* was not upregulated after herbivory by *Lymantria dispar*, a generalist caterpillar feeding on poplar. In contrast, *PtTPS19/20* transcript accumulation was slightly down regulated after herbivore damage (Fig. 7).

**Fig. 8:**
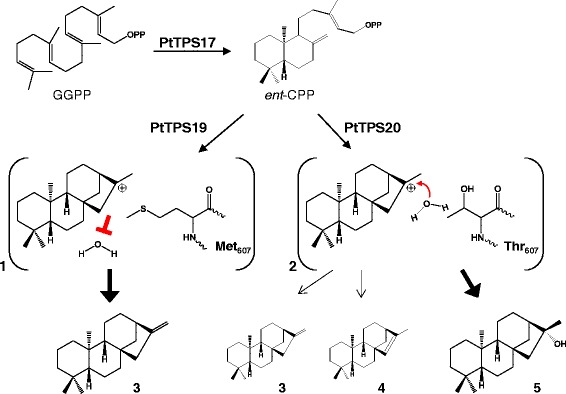
Putative reaction mechanism for *ent*-kaurene and 16α-hydroxy-*ent*-kaurane formation in poplar. The class II terpene synthase PtTPS17 catalyzes the conversion of GGPP into *ent*-CPP. Two highly similar class I enzymes, PtTPS19 and PtTPS20, accept *ent*-CPP as a substrate and convert it into *ent*-kaur-16-ene, the precursor for gibberellin biosynthesis or 16α-hydroxy-*ent*-kaurane, respectively. The product specificity seems to be controlled by one amino acid in the protein active center excluding (1) or allowing (2) the quenching of the beyeran-16-yl cation by a water molecule (modified from [33]). 3, *ent*-kaurene; 4, *ent*-isokaurene; 5, 16α-hydroxy-*ent*-kaurane

## Discussion

Labdane-related diterpenes are important plant metabolites and are known to function in primary as well as in secondary plant metabolism. Their formation starts with the cyclization of GGPP catalyzed by class II diterpene synthases. The resulting cyclic prenyldiphosphates are substrates for class I diterpene synthases which form the final diterpene hydrocarbons and alcohols. We showed that *P. trichocarpa* contains two putative class II diterpene synthases (PtTPS17/18) as well as two diterpene synthases (PtTPS19/20) with homology to class I enzymes. Heterologous expression in *E. coli* revealed that PtTPS17 catalyzed the conversion of GGPP into *ent*-CPP while the second putative class II enzyme PtTPS18 was inactive. PtTPS19 and PtTPS20 showed class I enzyme activity converting *ent*-CPP into *ent*-kaurene and 16α-hydroxy-*ent*-kaurane, respectively (Fig. 3, Additional file 1: Figure S2).

The tetracyclic *ent*-kaurene is a universal intermediate in the biosynthesis of gibberellins, important plant hormones controlling diverse growth processes such as germination, cell elongation and flowering [8]. Arabidopsis *ga1* (*ent-CPS*) mutants, for example, interrupted in *ent*-kaurene biosynthesis, show a male-sterile dwarfed phenotype [15, 28], indicating that *ent*-kaurene-derived gibberellins are essential for plant development and reproduction. *ent*-CPS and KS enzymes are found in all higher plants [29] and they have been identified and characterized from a number of mainly herbaceous species like rice and Arabidopsis [15, 16, 25]. The enzymes PtTPS17 and PtTPS19 characterized in this work produce *ent*-CPP and *ent*-kaurene, respectively, and are most likely the key enzymes for gibberellin biosynthesis in poplar. Thus, their identification and characterization provide a basis for further studies about gibberellin formation, regulation and function in this fast growing, woody perennial plant species.

The duplication of genes involved in primary metabolism and subsequent sub- or neofunctionalization of the resulting copies is believed to drive the evolution of plant secondary metabolism [30]. In general, plant CPS and KS are encoded by single copy genes [13]. However, in a few plant species, gene duplication led to an expansion of the *CPS* and *KS* gene families. In these plants, one *CPS* gene and one *KS* gene retained their functions in gibberellin biosynthesis [25]. Rice, for example, contains three *CPS*-like genes and ten *KS*-like genes in addition to the single *CPS*/*KS* gene pair [31], and it has been shown that most of these *CPS/KS*-like genes were recruited for the formation of secondary compounds such as diterpenoid phytoalexins. In poplar, a recent genome duplication event and a recent tandem gene duplication gave rise to two copies of the *CPS* and *KS* genes, respectively ([22], Additional file 1: Figure S1). Presumably, subsequent mutations led to the inactivation of one of the *CPS* gene copies while the *KS* gene *PtTPS20* evolved new product specificity. Thus, *PtTPS19* and *PtTPS20* likely represent an example for the evolution of a gene involved in secondary metabolism from an ancestor that functions in primary metabolism.

Both PtTPS19 and PtTPS20 are highly similar on the amino acid level but instead of producing only *ent*-kaurene, PtTPS20 produced mainly 16α-hydroxy-*ent*-kaurane and small amounts of *ent*-kaurene and *ent*-isokaurene (Fig. 3). While the production of alcohols is quite common for mono- and sesquiterpene synthases, the vast majority of diterpene synthases produce hydrocarbons and reports of diterpene synthases producing alcohols are rare. One example is the bifunctional diterpene synthase from *Picea abies* producing the thermally unstable hydroxyabietene as its primary product [32]. To our knowledge, the only diterpene synthase described to produce 16α-hydroxy-*ent*-kaurane is the bifunctional PpCPS/KS from the bryophyte *Physcomitrella patens* [14].

It was postulated that the production of 16α-hydroxy-*ent*-kaurane results from a quenching of the beyeran-16-yl cation through the addition of a water molecule instead of double bond formation via a simple deprotonation [14]. Modeling the three-dimensional structures of PtTPS19 and PtTPS20 enabled us to identify one amino acid in the active site which determines the product specificity of the enzymes (Fig. 5). The conversion of methionine 607 into threonine in PtTPS19 resulted in a product profile nearly identical to that of PtTPS20 and the complementary exchange of threonine 607 into methionine in PtTPS20 completely transformed the enzyme into a KS like PtTPS19 (Fig. 5). The larger methionine side chain of PtTPS19 likely shields the carbocation of the beyeran-16-yl intermediate and thus prevents the addition of a water molecule (Fig. 8). In contrast, the smaller, more polar threonine residue might form a water-binding pocket and/or change the substrate conformation, thus allowing the addition of a water molecule (to give 16α-hydroxy-*ent*-kaurane) as well as proton abstraction at two different positions (to give *ent*-kaurene and *ent*-isokaurene). However, the hydroxyl group of the threonine side chain seems not to be involved in the coordination of the water molecule as the replacement of threonine with alanine did not change the product specificity. A similar effect was already observed for PpCPS/KS which produces 16α-hydroxy-*ent*-kaurane and smaller amounts of *ent*-kaurene [14, 33]. Kawaide and coworkers (2011) could identify an alanine residue determining the product specificity of the enzyme. An exchange of alanine 710, which is located at the corresponding position to threonine/methionine 607 in PtTPS19/20, into methionine or an amino acid residue with a larger hydrophobic side chain led to an enzyme able to produce only *ent*-kaurene, while other smaller hydrophilic side chains in this position still allowed the production of the alcohol. These findings indicate that this amino acid position plays a role in forming the active site cavity rather than being involved in water binding as described, for example, for an asparagine in the active site of a 1,8-cineole synthase from *Salvia fruticosa* [34]. Interestingly, the amino acid corresponding to threonine 607 in PtTPS20 is strongly conserved as a methionine in KS of higher plants [33]. Like for PtTPS19, the exchange of this conserved methionine into a smaller alanine in the bifunctional KS of white spruce (*Picea glauca*) led to the production of the alcohol 16α-hydroxy-*ent*-kaurane and smaller amounts of *ent*-isokaurene [35]. However, the mutated spruce enzyme only produced 40 % 16α-hydroxy-*ent*-kaurane, retaining most of its original activity in producing *ent*-kaurene, while in poplar the single amino acid switch had a stronger impact on KS activity. The fact that the exchange of few amino acids can specifically alter diterpene synthase product outcome is well known [30, 33, 35, 36]. In rice, for example, the alteration of a single amino acid was sufficient to convert an isokaurene synthase into a pimaradiene synthase [30].

The CPS enzymes involved in gibberellin biosynthesis are in general characterized by a highly conserved histidine residue which leads to an inhibition of enzyme activity at higher Mg^2+^ concentrations [24, 37]. This effect has been hypothesized to be a mechanism for controlling the flux of *ent*-CPP into gibberellin biosynthesis [24, 37]. Although the *ent*-CPP synthase PtTPS17 possesses this conserved histidine and *ent*-CPP formation is inhibited at higher Mg^2+^ concentrations (Fig. 4), the similar expression pattern of *PtTPS17* and *PtTPS19/20* indicates that PtTPS17 provides the substrate for both *ent*-kaurene as well as 16α-hydroxy-*ent*-kaurane formation (Figs. 2, 4, 6). Due to their general growth promoting function, *CPS* and *KS* genes are reported to be constitutively expressed in different plant organs with the highest expression in rapidly growing tissues and lower expression in fully expanded leaves and roots while the abundance of diterpene genes for secondary metabolism is more restricted [25, 38–41]. Although *PtTPS17* and *PtTPS19/20* were expressed in all tested poplar tissues, the highest transcript accumulation was found in roots. However, sequencing of *PtTPS19/20* transcripts revealed that the *KSL* gene *PtTPS20* accounted for more than 60 % of measured transcripts in roots (Fig. 6), indicating a specific production of 16α-hydroxy-*ent*-kaurane in this organ, probably supported by a high abundance of PtTPS17 to generate the precursor *ent*-CPP. A similar phenomenon was observed in *Stevia rebaudiana.* In this plant the expression of *ent*-*CPS* and a duplicated *KS* gene was found to be highest in mature leaves which was opposite to gibberellin biosynthesis, and both genes were concluded to be involved in steviol glycoside biosynthesis [39].

Unfortunately, our attempts to measure TPS enzyme activity in crude poplar protein extracts failed. Thus we were not able to compare in vivo enzyme activity with gene expression data. However, as a multitude of studies have shown that the in vitro product profiles as well as expression patterns of terpene synthases usually correlate well with the terpenes produced by the respective plants [20, 26, 42], it is likely that diterpene synthase activity in poplar is also reflected by *TPS* transcript accumulation.

The role of 16α-hydroxy-*ent*-kaurane in poplar remains unclear. The moss *P. patens* releases this diterpene alcohol as a volatile at a high rate, but nothing is known about its function [43]. As the terpene synthase gene *PtTPS20* seems to be constitutively expressed in poplar, the 16α-hydroxy-*ent*-kaurane could function as an allelochemical or phytoanticipin. However, as we could not detect this compound in hexane extracts of plant material, the alcohol could also be the precursor for other yet unidentified compounds in poplar. *Ent*-isokaurene, for example, which is also produced by PtTPS20 is a putative intermediate in the biosynthesis of oryzalide A, an antimicrobial compound found in rice leaves [31]. However, the diterpenoid alcohol 16α-hydroxy-*ent*-kaurane might also act as a signaling molecule as was already demonstrated for a bicyclic diterpenoid alcohol in tobacco which mediates the activation of defense responses in tobacco (Seo, 2003).

## Conclusion

We identified an *ent*-CPS and a KS in poplar that appear to be involved in gibberellin biosynthesis. The *KS* gene seems to have undergone a recent tandem gene duplication and sub-/neofunctionalization accompanied by a single amino acid change that was sufficient to turn the KS into a KSL. By allowing the quenching of the beyeran-16-yl cation through the addition of a water molecule, the major product was altered from *ent*-kaurene to 16α-hydroxy-*ent*-kaurane. While genes for gibberellin biosynthesis seem to be expressed constitutively in all organs, the *KSL* gene was highly abundant in roots indicating a possible function in specialized metabolism.

## Methods

### Plant and insect material

Western balsam poplar (*Populus trichocarpa*) trees were propagated from monoclonal stem cuttings (clone 625, NW-FVA, Hann. Münden, Germany) and grown under summer conditions in the greenhouse (24 °C, 60 % rel. humidity, 16 h/8 h light/dark cycle) in a 1:1 mixture of sand and soil (Klasmann potting substrate, Klasmann-Deilmann, Geeste, Germany), until they reached about 1 m in height. Leaves were numbered according to the leaf plastochron index (LPI) [44]. LPI 2 to LPI 7 leaves, the stem in between these leaves, as well as poplar roots were harvested. Additionally, stem cuttings were planted and just opened leafbuds were harvested after 13 days. Herbivore-treated plant material was obtained as described in [45]. Briefly, trees were infested with *L. dispar* larvae on one leave, enclosed with a PET bag (“Bratschlauch”, Toppits, Minden, Germany) by fixing the ends of the bags with cable binders. Five *L. dispar* caterpillars in third to fourth instar starved for 12 h were released on the leaves. The caterpillars were fed with *P. trichocarpa* leaves for one week prior to the onset of the experiment. Caterpillars were allowed to feed for 24 h (16.00 – 16.00 h). After harvesting, plant material was immediately flash-frozen with liquid nitrogen and stored at −80 °C until further processing.

Gypsy moth (*Lymantria dispar*) egg batches were kindly provided by Hannah Nadel, APHIS, USA. After hatching, the caterpillars were reared on an artificial diet (Gypsy moth diet, MP Biomedicals LLC, Illkirch, France).

### Plant tissue sampling, RNA extraction and reverse transcription

Plant material was ground in liquid nitrogen. The total RNA was isolated using an Invisorb Spin Plant RNA Mini Kit (Invitek GmbH, Berlin, Germany) according to the manufacturer’s instructions. RNA concentration, purity and quality were assessed using a spectrophotometer (NanoDrop 2000c, Thermo Scientific, Wilmington, USA) and an Agilent 2100 Bioanalyzer (Agilent Technologies GmbH, Waldbronn, Germany). Prior to cDNA synthesis, 0.75 μg RNA was DNase-treated using 1 μL DNase (Fermentas GmbH, St. Leon Roth, Germany). Single-stranded cDNA was prepared from the DNase-treated RNA using SuperScript^™^ III reverse transcriptase and oligo (dT_12-18_) primers (Invitrogen, Carlsbad, CA, USA).

### Identification and isolation of *KS(L)* and *CPS* genes

To identify putative *KS(L)* and *CPS* genes, a TBLASTN search was conducted with the *P. trichocarpa* genome database (http://www.phytozome.net/poplar) using AtCPS (Q38802) and AtKS (AAC39443) as query sequences. Two putative *KS(L)* and two putative *CPS* genes were identified in the genome and could be amplified from a cDNA pool obtained from *P. trichocarpa* leaves, stems, buds and roots*.* Primer sequence information is available in Additional file 1: Table S2. PCR products were cloned into the sequencing vector pCR®^-^Blunt II-TOPO® (Invitrogen) and both strands were fully sequenced. Signal peptide prediction was done using the TargetP 1.1 server (http://www.cbs.dtu.dk/services/TargetP/), TargetLoc (https://abi.inf.uni-tuebingen.de/Services/MultiLoc), and PSORT (http://psort.hgc.jp/form.html) (see Additional file 1: Table S3). Sequences were deposited in GenBank with the accession numbers KT877421 (*PtTPS17*), KT877422 (*PtTPS18*), KT877423 (*PtTPS19*), and KT877424 (*PtTPS20*).

### Heterologous expression of CPS and KS(L) in *E. coli*

For heterologous expression, genes were N-terminally truncated (PtTPS19/20: Δ42 aa; PtTPS17: Δ65 aa; PtTPS18: full length, Δ92 aa) and cloned into the bacterial expression vector pET200 (Invitrogen). Cultures of *E. coli* strain BL21(DE3) were grown at 37 °C and 220 rpm, placed at 18 °C and 180 rpm after reaching an OD_600_ = 0.5, induced with 1 mM IPTG 60 min later, and grown for another 18 h. The cells were collected by centrifugation (10 min, 5000 *g*), placed in chilled extraction buffer (50 mM Tris HCl, pH = 7.5, 10 % glycerol (v/v), 10 mM MgCl_2_, 5 mM dithiothreitol, 5 mM sodium ascorbate, 1× Protease inhibitor Mix HP (SERVA,Germany), 25U Benzonase Nuclease (Merck, Germany), and 0.2 mg/mL lysozyme), and disrupted by a 3 × 30 s treatment with a sonicator (Bandelin UW2070, Berlin, Germany; 50 %). Cell fragments were removed by centrifugation at 14,000 g (10 min, 4 °C) and the supernatant was either directly desalted into assay buffer (10 % glycerol (v/v), 10 mM TrisHCl pH = 7.5, 1 mM dithiothreitol) by passage through an Econopac 10DG column (BioRad, Hercules, CA, USA), or the protein was purified from the supernatant using Ni-NTA Spin Columns (Qiagen, Hilden, Germany) and subsequently desalted through an Illustra NAP-5 Column (GE Healthcare).

### Analysis of recombinant KS(L) and CPS

To determine the catalytic activity of CPS, enzyme assays containing 80 μL of the bacterial extract or purified protein and 20 μL assay buffer with 50 μM (*E,E,E*)-GGPP (Sigma, Germany) and 5 mM MgCl_2_, in a Teflon-sealed, screw-capped 1 ml GC glass vial were performed and overlaid with 100 μl hexane. KS and KSL activity was determined as described above by mixing 40 μL of CPS extract or purified protein with 40 μL KS/KSL extract or purified protein. After incubation for 2 h at 25 °C, the hexane phase was collected and analyzed using GC-MS. For analyzing the dependence of different Mg^2+^ concentrations on PtTPS17/18 activity, assays were set up as triplicates as described above with 0.1, 1 or 10 mM MgCl_2_ and 5 or 50 μM GGPP, incubated for 30 min (4 h for PtTPS18) and stopped by adding 20 μl 5 N HCl and extracted after 15 min.

As negative controls, we substituted the CPS or KS(L) extracts with raw protein extracts from *E. coli* expressing an empty vector control. These assays demonstrated that *E. coli* extracts converted GGPP unspecifically into geranyllinalool, but no additional diterpene production was supported (Fig. 3).

The KS(L)/CPS enzyme products were analyzed and identified using an Agilent 6890 Series gas chromatograph coupled to an Agilent 5973 quadrupole mass selective detector (interface temp, 250 °C; quadrupole temp, 150 °C; source temp, 230 °C; electron energy, 70 eV). The GC was operated with a DB-5MS column (Agilent, Santa Clara, USA, 30 m × 0.25 mm × 0.25 μm). 1 μl of the hexane samples was injected without split at an initial oven temperature of 80 °C. The temperature was held for 2 min, than increased to 280 °C with a gradient of 5 °C min^−1^, and further increased to 320 °C with a gradient of 100 °C min^−1^ and a hold of 1 min. Compounds were identified by comparison of retention times and mass spectra to those of reference spectra in the Wiley and National Institute of Standards and Technology libraries and in the literature (Joulain, 1998) and to those of authentic standards (*ent*-kaurene, 16α-hydroxy-ent-kaurane) which were kindly provided by Prof. Reuben Peters (Iowa State University, USA).

### Modeling and site-directed mutagenesis

To identify amino acids controlling the product specificity of PtTPS19 and PtTPS20, homology-based structure models of PtTPS19 and PtTPS20 were created using the SwissModel web service (swissmodel.expasy.org) and the crystal structure of abietadiene synthase from *Abies grandis* (PDB 3S9V) as template. Visualization of the model was done using Pymol (http://www.pymol.org/).

For site-directed mutagenesis, 30 ng pET200/D-TOPO® vector harboring the N-terminal truncated version of either *PtTPS19* or *PtTPS20* were used as template in a mutagenesis PCR (18 cycles, Phusion® High-Fidelity DNA Polymerase, New England Biolabs GmbH, Frankfurt, Germany), according to manufacturer’s instructions (for primer information see Additional file 1: Table S2). The template DNA was digested with *Dpn*I and the PCR product was inserted and amplified in *E. coli* TOP10 (Invitrogen).

### qRT-PCR analysis of *CPS and KS(L)* expression

cDNA was prepared as described above. For the amplification of *PtTPS17, PtTPS18 and PtTPS19/20* gene fragments with a length between 100 and 170 bp, gene specific primer pairs were designed having a T_m_ of about 60 °C, a GC content between 39 and 45 % and a primer length in the range of 20 – 25 nt (Additional file 1: Table S2). Due to the high sequence similarity of *PtTPS19* and *PtTPS20*, a primer pair specific for both sequences was used for amplification. Sequencing of PCR products obtained from three biological replicates of each poplar organ was used to determine the average percentage for the transcripts of *PtTPS19* and *PtTPS20*.

Primer specificity was confirmed by agarose gel electrophoresis, melting curve analysis and standard curve analysis, and by sequence verification of cloned PCR amplicons. *Ubiquitin* was used as a reference gene [46]. Samples were run in triplicates using Brilliant® III SYBR® Green QPCR Master Mix (Stratagene, CA, USA) with ROX as reference dye. The following PCR conditions were applied for all reactions: initial incubation at 95 °C for 3 min followed by 40 cycles of amplification (95 °C for 20 s, 60 °C for 20 s). Plate reads were taken during the annealing and the extension step of each cycle. Data for the melting curves were recorded at the end of cycling from 55 to 95 °C.

All samples were run on the same PCR machine (MxPro – Mx3000P, Stratagene, Agilent Technologies, USA) in an optical 96-well plate. Five biological replicates were analyzed as triplicates in the qRT-PCR for each of the three treatments. Data for the relative quantity to calibrator average (dRn) were exported from the MxPro Software.

### Plant terpene and protein extraction

For terpene extraction, 100 mg of tissue powder (leaf buds, leaves, stems or roots) was extracted with 500 μl hexane containing 20 ng/μl nonylacetate as an internal standard. The extraction was carried out for 4 h at room temperature with vigorous vortexing. The hexane phase was then removed and 2 μl of the hexane samples were injected without split on a GC-MS as described above, except a different temperature program was used. After an initial oven temperature of 45 °C, the temperature was held for 2 min, then increased to 270 °C with a gradient of 6 °C min^−1^, and further increased to 340 °C with a gradient of 60 °C min^−1^ and a hold of 2 min. We could identify several mono- and sesquiterpenes in these extracts, but no diterpenoid compounds were detected.

For crude plant protein extracts 1 ml ice-cold protein extraction buffer (100 mM potassium phosphate, pH 8.0, 5 mM dithiothreitol, 2 mM ethylenediaminetetraacetic acid, 1 % (w/v) polyvinylpyrrolidone (Mr = 10,000), 4 % (w/v) polyvinylpoly- pyrrolidone, 1 mM phenylmethylsulfonyl fluoride) was added to 100 mg of frozen, freshly ground poplar tissue and samples were incubated for 1 h (4 °C, 150 rpm), centrifuged (30 min at 4 °C, 12.000 × *g*) and desalted into assay buffer (via a Zeba™ Spin Desalting Column, 7 K MWCO, Thermo Scientific). Protein assays were conducted as described above using 45 μl desalted crude protein extract and analyzed using GC-MS as described above. No diterpene formation could be detected.

### Phylogenetic tree reconstruction

For the construction of a phylogenetic tree containing the characterized PtCPS and PtKS(L) enzymes and other representative CPS and KS enzymes, we used the MUSCLE algorithm (gap open, −2.9; gap extend, 0; hydrophobicity multiplier, 1.5; clustering method, upgmb) implemented in MEGA5 [47] to compute an amino acid alignment of CPS and KS(L) enzymes. Based on the MUSCLE alignment, the tree was reconstructed with MEGA5 using a neighbor-joining algorithm (Poisson model). A bootstrap resampling analysis with 1000 replicates was performed to evaluate the tree topology.

An alignment of PtCPS and PtKS(L) enzymes with the characterized CPS and KS from *A. thaliana* was constructed and visualized using BioEdit (http://www.mbio.ncsu.edu/bioedit/bioedit.html) and the ClustalW algorithm.

### Statistical analysis

To test for significant differences in gene expression between different poplar organs, log transformed data were analyzed using a one way analysis of variance (ANOVA) followed by a pairwise multiple comparison (Holm-Sidak method) using SigmaPlot 11.0 for Windows (Systat Software Inc. 2008).

## Additional file

Additional file 1:
**Figure S1.** *KS(L)* genes located on chromosome 8. **Figure S2**. GC-MS analysis of *ent*-CPS, *syn*-CPS, *n*-CPS and PtTPS17 products. **Table S1.** C_q_ values of poplar *CPS* and *KS(L)*. **Table S2.** Oligonucleotides used in this study. **Table S3.** Signalpeptide prediction using different prediction algorithms. (PPTX 147 kb)

## Abbreviations

CPS: Copalyl diphosphate synthase
CPP: Copalyl diphosphate
GGPP: Geranylgeranyl diphosphate
KS(L): Kaurene synthase(-like) enzymes
LPI: Leaf plastochron index
qRT-PCR: Quantitative real time PCR
TPS: Terpene synthase
