# Supplementary material for: One amino acid makes the difference: the formation of ent-kaurene and 16α-hydroxy-ent-kaurane by diterpene synthases in poplar
Source: BMC Plant Biol. 2015 Oct 28;15:262. doi: 10.1186/s12870-015-0647-6 (PMC4625925; doi:10.1186/s12870-015-0647-6)
Supplement: Additional file 1: — Figure S1. KS(L) genes located on chromosome 8. Figure S2. GC-MS analysis of ent-CPS, syn-CPS, n-CPS and PtTPS17 products. Table S1. Cq values of poplar CPS and KS(L). Table S2. Oligonucleotides used in this study. Table S3. Signalpeptide prediction using different prediction algorithms. (PPTX 147 kb) [file 12870_2015_647_MOESM1_ESM.pptx]

## Slide 1
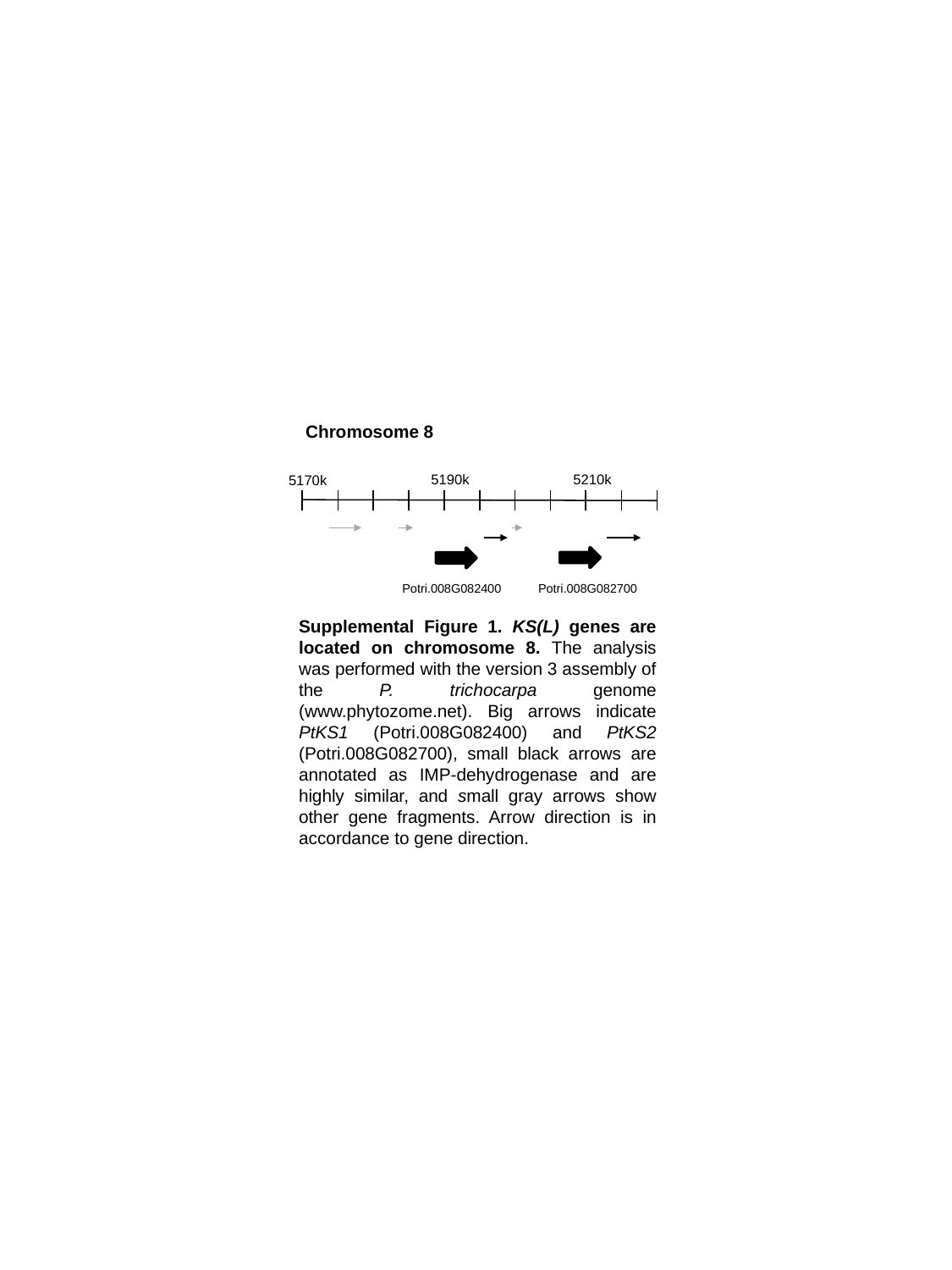

Chromosome 8
5190k
5210k
5170k
Potri.008G082400
Potri.008G082700
Supplemental Figure 1. KS(L) genes are located on chromosome 8. The analysis was performed with the version 3 assembly of the P. trichocarpa genome (www.phytozome.net). Big arrows indicate PtKS1 (Potri.008G082400) and PtKS2 (Potri.008G082700), small black arrows are annotated as IMP-dehydrogenase and are highly similar, and small gray arrows show other gene fragments. Arrow direction is in accordance to gene direction.

## Slide 2
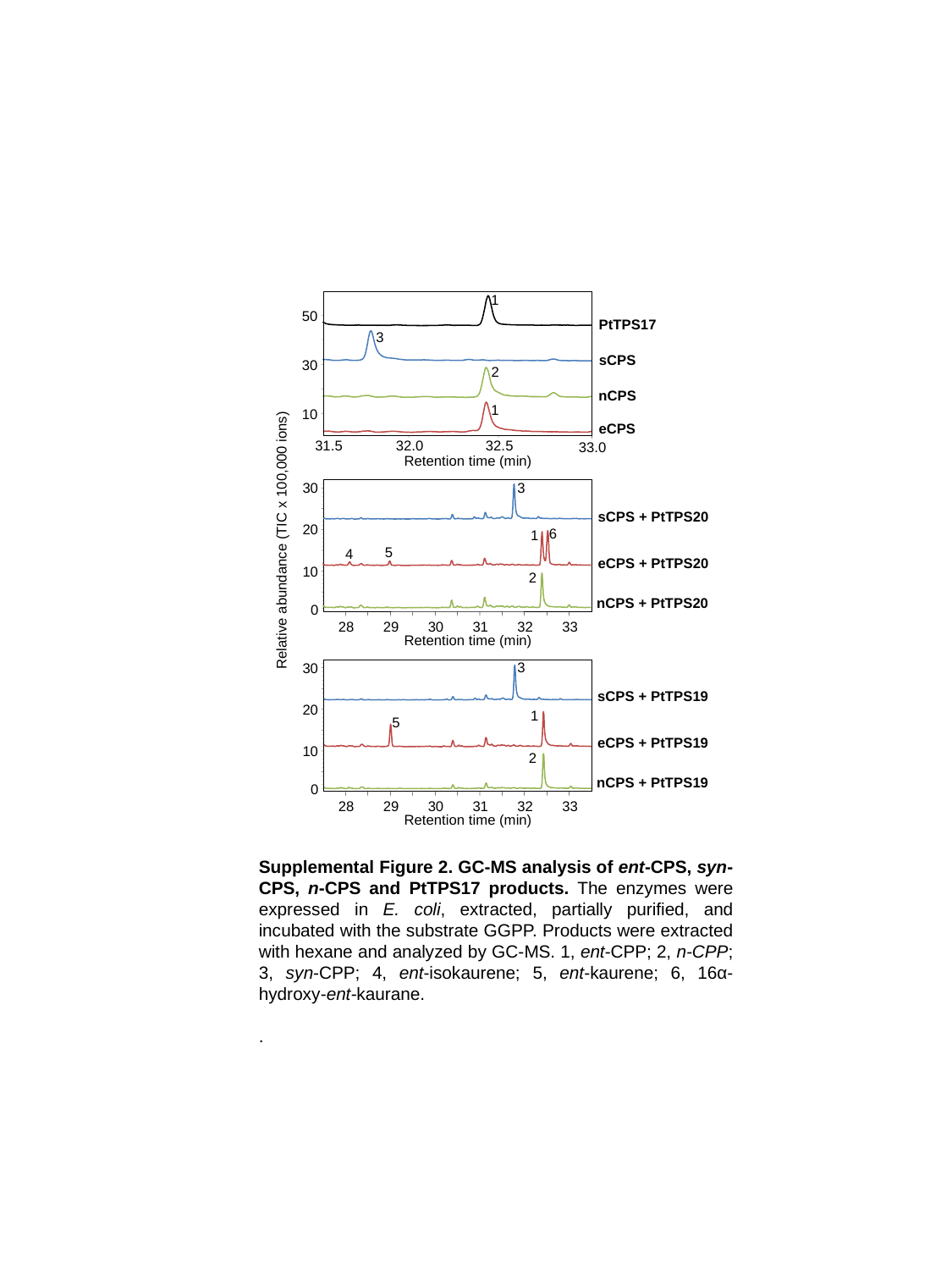

1
50
30
10
31.5
32.0
32.5
33.0
PtTPS17
3
sCPS
2
nCPS
1
eCPS
Retention time (min)
3
30
20
10
0
28
29
30
31
32
33
sCPS + PtTPS20
6
1
5
4
eCPS + PtTPS20
2
nCPS + PtTPS20
Relative abundance (TIC x 100,000 ions)
Retention time (min)
3
30
20
10
0
28
29
30
31
32
33
sCPS + PtTPS19
1
5
eCPS + PtTPS19
2
nCPS + PtTPS19
Retention time (min)
Supplemental Figure 2. GC-MS analysis of ent-CPS, syn-CPS, n-CPS and PtTPS17 products. The enzymes were expressed in E. coli, extracted, partially purified, and incubated with the substrate GGPP. Products were extracted with hexane and analyzed by GC-MS. 1, ent-CPP; 2, n-CPP; 3, syn-CPP; 4, ent-isokaurene; 5, ent-kaurene; 6, 16α-hydroxy-ent-kaurane.
.

## Slide 3
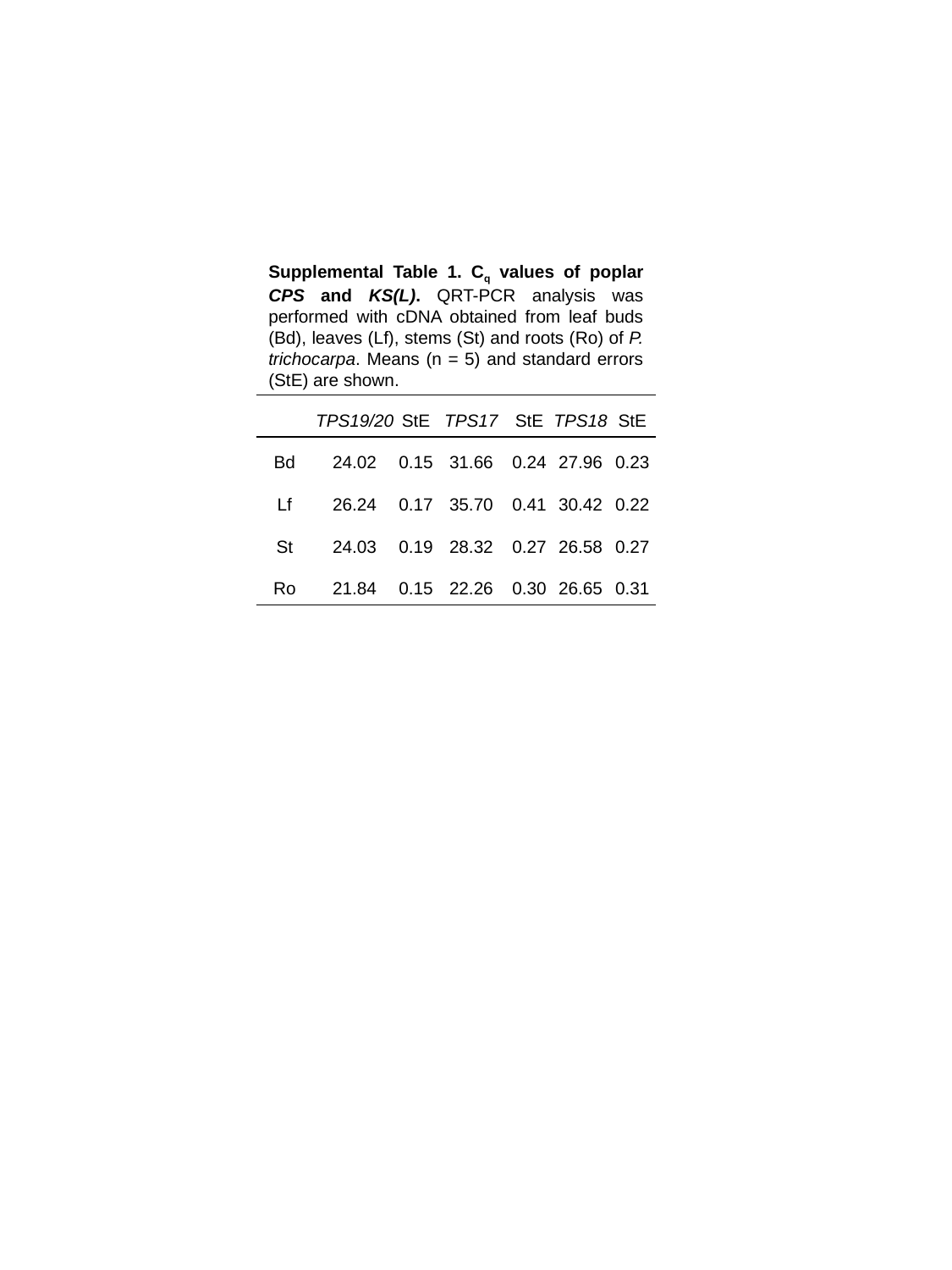

Supplemental Table 1. Cq values of poplar CPS and KS(L). QRT-PCR analysis was performed with cDNA obtained from leaf buds (Bd), leaves (Lf), stems (St) and roots (Ro) of P. trichocarpa. Means (n = 5) and standard errors (StE) are shown.
| | TPS19/20 | StE | TPS17 | StE | TPS18 | StE |
| --- | --- | --- | --- | --- | --- | --- |
| Bd | 24.02 | 0.15 | 31.66 | 0.24 | 27.96 | 0.23 |
| Lf | 26.24 | 0.17 | 35.70 | 0.41 | 30.42 | 0.22 |
| St | 24.03 | 0.19 | 28.32 | 0.27 | 26.58 | 0.27 |
| Ro | 21.84 | 0.15 | 22.26 | 0.30 | 26.65 | 0.31 |

## Slide 4
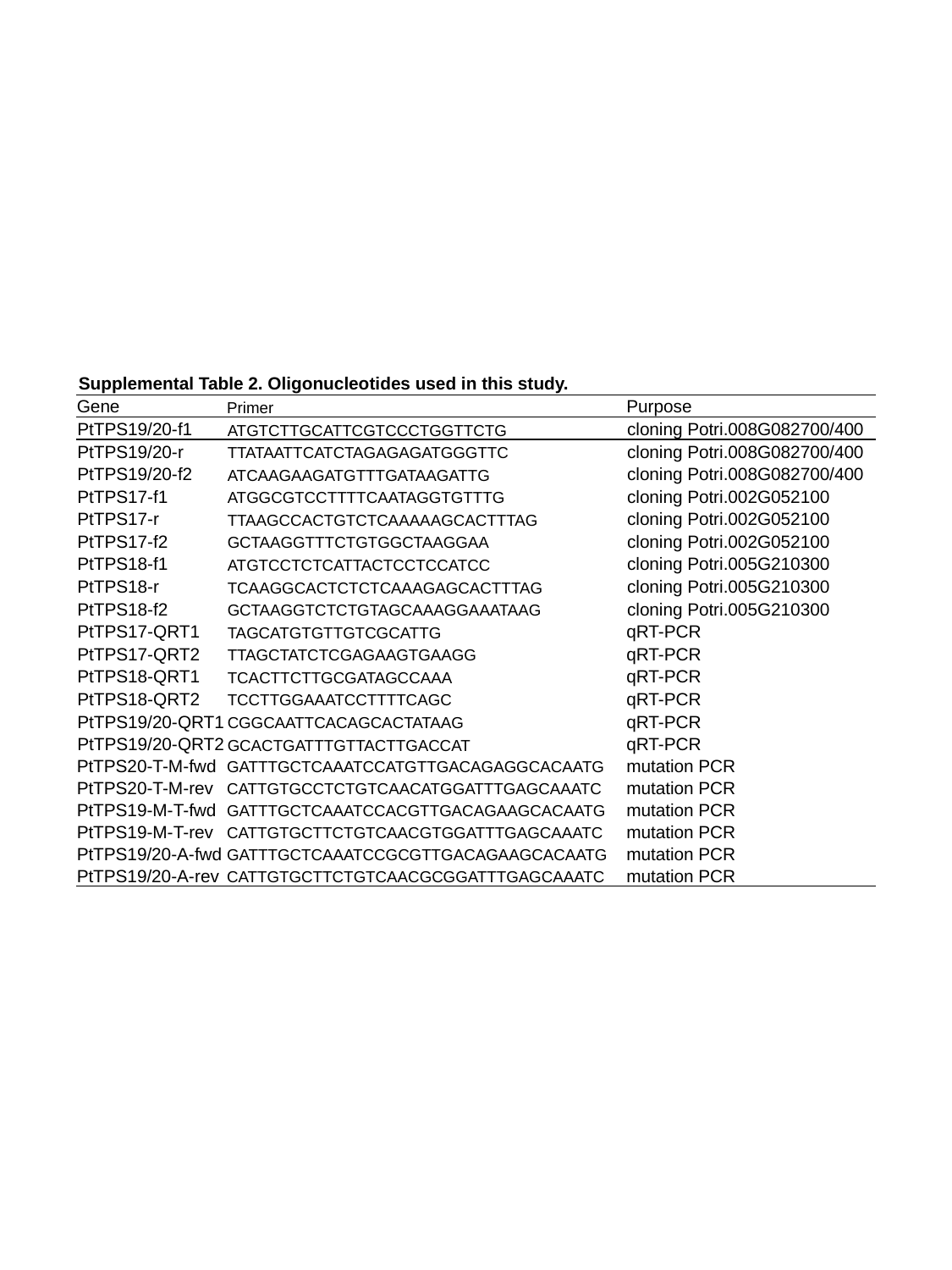

Supplemental Table 2. Oligonucleotides used in this study.
| Gene | Primer | Purpose |
| --- | --- | --- |
| PtTPS19/20-f1 | ATGTCTTGCATTCGTCCCTGGTTCTG | cloning Potri.008G082700/400 |
| PtTPS19/20-r | TTATAATTCATCTAGAGAGATGGGTTC | cloning Potri.008G082700/400 |
| PtTPS19/20-f2 | ATCAAGAAGATGTTTGATAAGATTG | cloning Potri.008G082700/400 |
| PtTPS17-f1 | ATGGCGTCCTTTTCAATAGGTGTTTG | cloning Potri.002G052100 |
| PtTPS17-r | TTAAGCCACTGTCTCAAAAAGCACTTTAG | cloning Potri.002G052100 |
| PtTPS17-f2 | GCTAAGGTTTCTGTGGCTAAGGAA | cloning Potri.002G052100 |
| PtTPS18-f1 | ATGTCCTCTCATTACTCCTCCATCC | cloning Potri.005G210300 |
| PtTPS18-r | TCAAGGCACTCTCTCAAAGAGCACTTTAG | cloning Potri.005G210300 |
| PtTPS18-f2 | GCTAAGGTCTCTGTAGCAAAGGAAATAAG | cloning Potri.005G210300 |
| PtTPS17-QRT1 | TAGCATGTGTTGTCGCATTG | qRT-PCR |
| PtTPS17-QRT2 | TTAGCTATCTCGAGAAGTGAAGG | qRT-PCR |
| PtTPS18-QRT1 | TCACTTCTTGCGATAGCCAAA | qRT-PCR |
| PtTPS18-QRT2 | TCCTTGGAAATCCTTTTCAGC | qRT-PCR |
| PtTPS19/20-QRT1 | CGGCAATTCACAGCACTATAAG | qRT-PCR |
| PtTPS19/20-QRT2 | GCACTGATTTGTTACTTGACCAT | qRT-PCR |
| PtTPS20-T-M-fwd | GATTTGCTCAAATCCATGTTGACAGAGGCACAATG | mutation PCR |
| PtTPS20-T-M-rev | CATTGTGCCTCTGTCAACATGGATTTGAGCAAATC | mutation PCR |
| PtTPS19-M-T-fwd | GATTTGCTCAAATCCACGTTGACAGAAGCACAATG | mutation PCR |
| PtTPS19-M-T-rev | CATTGTGCTTCTGTCAACGTGGATTTGAGCAAATC | mutation PCR |
| PtTPS19/20-A-fwd | GATTTGCTCAAATCCGCGTTGACAGAAGCACAATG | mutation PCR |
| PtTPS19/20-A-rev | CATTGTGCTTCTGTCAACGCGGATTTGAGCAAATC | mutation PCR |

## Slide 5
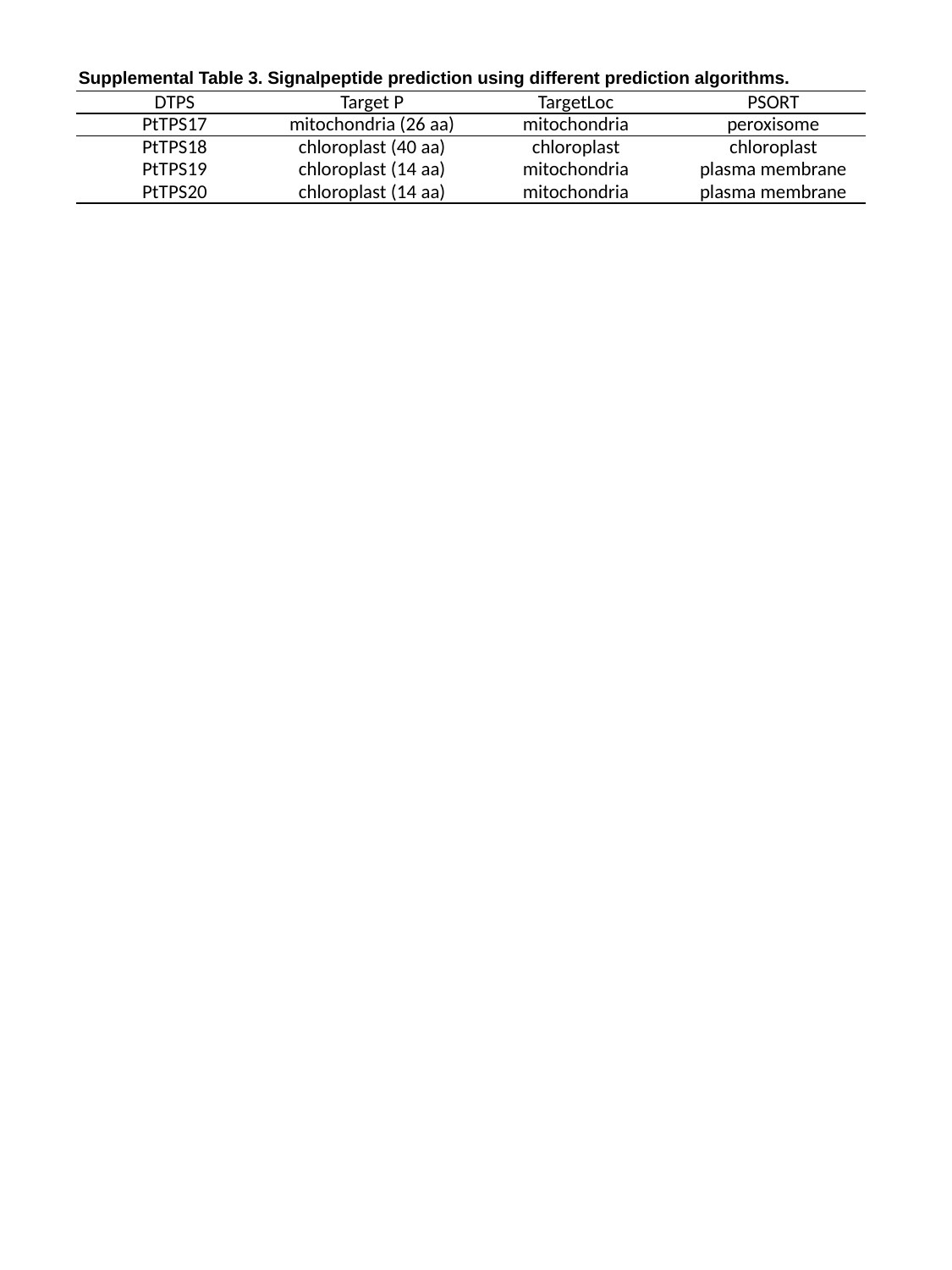

Supplemental Table 3. Signalpeptide prediction using different prediction algorithms.
| DTPS | Target P | TargetLoc | PSORT |
| --- | --- | --- | --- |
| PtTPS17 | mitochondria (26 aa) | mitochondria | peroxisome |
| PtTPS18 | chloroplast (40 aa) | chloroplast | chloroplast |
| PtTPS19 | chloroplast (14 aa) | mitochondria | plasma membrane |
| PtTPS20 | chloroplast (14 aa) | mitochondria | plasma membrane |
